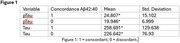# Discordance between CSF amyloid beta and PET‐derived amyloid status and its clinical implications

**DOI:** 10.1002/alz70856_106931

**Published:** 2026-01-22

**Authors:** Gemma Natalie Wright, James Neil Dodds, Rifa Sanjida Punnota, Roberto Vicidomini, Paul Edison

**Affiliations:** ^1^ Imperial College London, London, London, United Kingdom; ^2^ Imperial College London, London, Greater London, United Kingdom; ^3^ Division of Neurology, Department of Brain Sciences, Imperial College London, United Kingdom, London, London, United Kingdom

## Abstract

**Background:**

There has been a recent focus within Alzheimer's Disease (AD) research on potential CSF biomarkers as diagnostic tools. PET imaging and CSF biomarkers have been used to determine the amyloid status of individuals when characterising AD. While these measures have largely correlated with each other, it is also suggested that there could be patients who can have discordant results. However, it is still unclear what proportion of AD subjects demonstrate the discordance. Additionally, whether these changes are associated with different pathological and clinical characteristics of the patients. Here, we investigated the discordance between CSF Aβ42:40 and amyloid status and evaluated whether there are any significant changes in their levels of tau aggregation.

**Method:**

313 participants’ data were selected from the ADNI database. The cutoff for Aβ42:40 was then calculated using the Youden Index resulting from receiver operating characteristic (ROC) curve. The formula used was *J* = max*c* {Se (*c*) + Sp (*c*) − 1}, resulting in a maximum cutoff of *J* = 0.057 for Aβ42:40. This cutoff produced a discordance rate of 10.5%, with *n* = 33 of 313 patients being discordant. We then performed t‐tests to investigate the potential clinical differences between concordant and discordant patients.

**Result:**

Independent samples t‐tests indicated that levels of pTau181 and total Tau significantly differed between the concordant and discordant groups. pTau181 was significantly lower in the discordant group (t(71.2)=‐3.166, *p* = .002), as was Tau (t(53.9)=‐2.046, *p* = .046). Further data is found in Figure 1.

**Conclusion:**

Demonstration of different levels of tau deposition in the discordant groups implies there may be other pathological processes influencing neurodegeneration in these subjects. A discordance of 10% between CSF amyloid and PET amyloid implies that we should evaluate these subjects in greater detail before enrolling them in intervention studies.

**References**

1. Hansson, O. et al. (2019). https://doi.org/10.1186/s13195‐019‐0485‐0

2. Pyun, JM. et al. (2024) https://doi.org/10.1038/s41398‐024‐02766‐6